# A Multi-Band Temperature Measurement Data Retrieval Method Based on the Chaotic Artificial Hummingbird Algorithm (CAHA)

**DOI:** 10.3390/s26072210

**Published:** 2026-04-02

**Authors:** Wenxiang You, Xiaojian Hao, Rui Jia, Pan Pei, Shenxiang Feng, Xining Wang

**Affiliations:** School of Instrument and Electronics, North University of China, Taiyuan 030000, China; sz202306054@st.nuc.edu.cn (W.Y.); peipan@nuc.edu.cn (P.P.); b20240602@st.nuc.edu.cn (S.F.); b20230622@st.nuc.edu.cn (X.W.)

**Keywords:** multispectral radiation thermometry, chaotic artificial hummingbird algorithm, temperature retrieval

## Abstract

To address the challenges of data processing caused by uncertain emissivity in multispectral radiation thermometry, this paper proposes a temperature retrieval method based on the Chaotic Artificial Hummingbird Algorithm (CAHA). Without relying on an assumed emissivity model, the method can automatically identify the emissivity distribution and selects the optimal output through multiple iterations to enhance accuracy. Simulations and offline tests conducted on rocket nozzles demonstrate that CAHA maintains high accuracy both in noise-free conditions and under 5% noise, with a single execution time of approximately 0.15 s. Furthermore, the method is validated through experiments on blackbody sources and candle flames: the relative error in retrieved temperature for blackbody sources remains below 0.93%, while the retrieved outer flame temperature of candle flames shows a relative error of 0.66% compared with thermocouple measurements. Combining high precision with rapid computation, this method is suitable for practical applications in radiation thermometry.

## 1. Introduction

Multispectral temperature measurement has been widely applied in metal processing industry [1,2,3,4], aerospace industry [5,6], gas turbine blade temperature monitoring [7,8] and other fields due to its non-contact nature, wide adaptability and high precision. However, the temperature measurement accuracy in practical engineering is restricted by the spectral emissivity of the object to be measured [9], and the emissivity of the object is limited by many factors such as temperature, material, and surface roughness of the object [10]. Therefore, it is extremely difficult to accurately solve the emissivity [11,12], and thus the multispectral temperature measurement method that does not rely on the assumed emissivity model has become the mainstream research direction in recent years. In the secondary measurement method, the reference temperature model for directly solving the emissivity and temperature without assuming the emissivity model was first proposed by J. Dai [13] et al. They used the genetic algorithm to convert the reference temperature mathematical model for rocket plume temperature measurement into a constrained optimization problem of the multi-band spectral average temperature difference function, with an accuracy of an error within 0.2%; S Gao [14] et al. used the genetic algorithm to solve the constrained optimization problem of the target to be measured in the high-temperature reflection environment for blade temperature measurement, with the maximum measurement error reduced from 4.16% to 1.20%.

J. Xing et al. respectively used Gradient Projection (GP), Internal Penalty Function (IPF), and Generalized Inverse Matrix-Exterior Penalty Function (GIM-EPF) [15,16,17] algorithms to directly solve the emissivity of the reference temperature mathematical model and inversely calculate the temperature, and tested it through the experimental data of dynamic measurement of rocket nozzles. The accuracy was reduced from the relative error of 1.5% or less of GP to the relative error of 1% or less of IPF, and then to the relative error of 0.6% or less of GIM-EPF. However, the implementation of the constraint optimization methods such as GP, IPF, and GIM-EPF depends on the appropriate initial emissivity solution, and it is necessary to preset the emissivity search range manually. Yu [18] et al. utilized the gradient of the average temperature difference function and calculated the optimal solution through the quasi-Newton algorithm BFGS. However, the accuracy of the BFGS optimization result also depends on the selection of the initial solution. Different choices of the initial solution will lead to significant changes in the accuracy of the optimization result. Zhao [19] et al. applied the meta-heuristic algorithm IGWO to the multi-band temperature inversion optimization problem, which controlled the relative error within 0.25% and reduced the single calculation time to 0.28 s. Although the meta-heuristic algorithm runs fast and has high result accuracy, due to the random generation characteristic of the initial population, it is prone to cause unstable result accuracy.

This paper presents a multi-band data inversion optimization method based on chaotic artificial hummingbird algorithm. This method initializes the solution population automatically through chaotic mapping and takes the optimal solution after multiple operations to achieve higher accuracy. Moreover, due to the low time complexity of the chaotic artificial hummingbird algorithm, its single operation time is only 0.15 s, and the time for 30 operations is only 4.5 s. This method can be applied to various practical engineering projects.

## 2. Principle of Algorithm

### 2.1. Reference Temperature Model

For a multi-wavelength thermometer with n channels, the output signal *V_λi_* of the i-th channel can be expressed as:

(1) $$V_{\lambda_{i}}=A_{\lambda_{i}}\varepsilon \left(\lambda_{i},T\right)\frac{1}{\lambda_{i}^{5}\left(e^{c_{2}/\lambda_{i}T}-1\right)}\left(i=1,2,\ldots ,n\right),$$

where *A_λi_* is the calibration coefficient that varies with wavelength, which is related to the detector’s own geometry, transmittance and response sensitivity, and has nothing to do with temperature. *T* is the true temperature of the measured target. *λ_i_* is the effective wavelength of the i-th channel. *ε*(*λ_i_*,*T*) is the spectral emissivity corresponding to the wavelength of *λ_i_* at the temperature of *T*. *C*_1_ and *C*_2_ are the first and second radiation constants respectively.

From Wien’s formula, it can be obtained that:

(2) $$V_{\lambda_{i}}=A_{\lambda_{i}}\varepsilon \left(\lambda_{i},T\right)\lambda_{i}^{-5}e^{-\frac{c_{2}}{\lambda_{i}T}}\left(i=1,2,\ldots ,n\right).$$

It should be noted that the application condition of Wien’s formula here is *C*_2_/*λ_i_T* >> 1.

The reference temperature model eliminates the coefficient *A_λi_* by pre-calibrating the standard blackbody temperature Tb. When the standard blackbody temperature is set as Tb, the output signal *V_λib_* of the i-th channel of the multispectral thermometer can be expressed as:

(3) $$V_{\lambda_{ib}}=A_{\lambda_{i}}\varepsilon \left(\lambda_{ib},T\right)\lambda_{i}^{-5}e^{-\frac{c_{2}}{\lambda_{ib}T}},$$

where ε(*λ_ib_, T*) is the spectral emissivity of a reference blackbody, which can be regarded as 1.

The ratio of Equations (2) and (3) is described by:

(4) $$\frac{V_{\lambda_{i}}}{V_{\lambda_{ib}}}=\varepsilon \left(\lambda_{i},T\right)e^{\frac{c_{2}}{\lambda_{i}}\left(\frac{1}{T_{b}}-\frac{1}{T}\right)}.$$

According to Equation (4), when *ε*(*λ_i_*,*T*) is determined, the inversion temperature *T_i_* of each channel of the multispectral pyrometer should be the same and infinitely close to the true temperature. Therefore, the temperature deviation of each channel temperature *T_i_* should also be infinitely close to 0. The mathematical expression should be:

(5) $$\sum_{i=1}^{n}{\left[T_{i}-E\left(T_{i}\right)\right]}^{2}=0,$$

where *E*(*T_i_*) is the average temperature of each channel, that is:

(6) $$E\left(T_{i}\right)=\frac{1}{n}\sum_{i=1}^{n}T_{i}^{2}.$$

From Equations (5) and (6), the target optimization function min*F* can be derived:

(7) $$\min F=\sum_{i=1}^{n}{\left[T_{i}-E\left(T_{i}\right)\right]}^{2}\to 0.$$

Taking the logarithm on both sides of Equation (4) yields:

(8) $$\ln\left(\frac{V_{\lambda_{i}}}{V_{\lambda_{ib}}}\right)=\ln\varepsilon \left(\lambda_{i},T\right)+\frac{c_{2}}{\lambda_{i}}\left(\frac{1}{T_{b}}-\frac{1}{T}\right).$$

Order $y_{i}=\frac{V_{\lambda_{i}}}{V_{\lambda_{ib}}}$, $x_{i}=\varepsilon \left(\lambda_{i},T\right)$, $a_{i}=\frac{c_{2}}{\lambda_{i}}$, $D_{i}=\frac{a_{i}}{T_{b}}-\ln y_{i}$, the temperature *T_i_* of each channel and the average temperature *E*(*T_i_*) of all channels can be expressed as:

(9) $$T_{i}=\frac{a_{i}}{\ln x_{i}+D_{i}},$$

(10) $$E(T_{i})=\frac{1}{n}\sum_{i=1}^{n}\left(\frac{a_{i}}{\ln x_{i}+D_{i}}\right).$$

Since the spectral emissivities of most materials range between 0.1 and 0.9, the emissivity can be constrained:

(11) $$\left\{\begin{array}{c} \varepsilon \left(\lambda_{i},T\right)\geq 0.1\\ -\varepsilon \left(\lambda_{i},T\right)\geq -0.9 \end{array}\right..$$

By restricting the range of emissivity, the above-mentioned multi-band temperature measurement data inversion can be transformed into a constrained optimization problem, which is in the following form:

(12) $${\left\{\begin{array}{c} \min F=\sum_{i=1}^{n}\left[\frac{a_{i}}{\ln x_{i}+D_{i}}-\frac{1}{n}\sum_{i=1}^{n}\left(\frac{a_{i}}{\ln x_{i}+D_{i}}\right)\right]\\ Ax_{i}\geq b \end{array}\right.}^{2}.$$

From the above expression, it can be seen that this model has transformed the temperature inversion process into a non-linear constrained optimization problem, which can be solved by a suitable optimization algorithm. The chaotic artificial hummingbird algorithm (CAHA) has great performance in the optimization problem of multi-extreme value functions. The CAHA proposed in this paper is very suitable for solving this problem.

### 2.2. Chaotic Artificial Hummingbird Algorithm

The Artificial Hummingbird Algorithm (AHA) is an intelligent optimization algorithm inspired by the foraging and flight behavior patterns of hummingbirds in nature. AHA simulates the foraging behavior of hummingbirds through three flight skills. And it sets up a visit table mechanism to record the visitation of each food source by the hummingbird and to guide the hummingbird in choosing the food source to visit. High-quality food sources will have more visitation opportunities.

The chaotic artificial hummingbird algorithm (CAHA) is an improvement upon the Artificial Hummingbird Algorithm (AHA). This algorithm introduces chaotic mapping to increase the diversity of the initial population and enhance the search capability for the global optimum. The selection of an appropriate chaotic map is crucial for achieving this improvement. According to recent research on chaotic optimization [20], different chaotic maps exhibit distinct invariant probability measures and traversal efficiencies. Among them, the Tent map possesses a uniform distribution characteristic with an invariant probability measure of f* = 1, achieving the highest traversal efficiency within the solution space. This property enables the initial population to cover the search space more uniformly, thereby improving the algorithm’s exploration capability and reducing the risk of premature convergence. Consequently, CAHA employs the Tent map for population initialization. The algorithm procedure of CAHA is introduced as follows.

(1) Initialize the algorithm parameters and the Visit Table, where the maximum number of iterations is *T_max* = 100 and the number of initial solutions is *Npop* = 50; the upper bound *Ub* of the initial solution is set to 0.9 and the lower bound *Lb* is 0.1.
(2) The flight coefficients *C*_1_ and *C*_2_ are randomly generated through Tent mapping.
(3) The flight type and foraging strategy of this flight are determined by the flight coefficients *C*_1_ and *C*_2_. The three flight skills are Axial flight, Diagonal flight, and Omnidirectional flight, respectively. The foraging strategies are divided into Guided foraging, Territorial foraging, and Migration foraging.
(4) If no superior food source is discovered in this round of foraging, conduct Chaotic traversal flight. The mathematical model of Chaotic traversal flight is:
  (13) $$v_{i}\left(t+1\right)=H_{i}\left(t,d\right)\cdot \left(Ub-Lb\right)/\left(N-2+2\times rand\right)\cdot D_{t}\cdot x_{i}\left(t\right)+x_{i}\left(t\right),$$
  where *H_i_*(*t*,*d*) represents the d-dimensional Tent chaotic vector produced by the i-th hummingbird in the d-dimensional solution space at time *t*
(5) Update the Visit Table in accordance with the foraging circumstances of this round.
(6) If *mod*(*t*, 2*Npop*) = 0 (where *t* denotes the current iteration number), Migration foraging is implemented. The hummingbird at the food source with the poorest grouting rate will migrate to a new food source randomly generated in the entire search space. The mathematical model of Migration foraging is:
  (14) $$x_{wor}\left(t+1\right)=Lb+T^{t}\left(i,d\right)\cdot \left(Ub-Lb\right),$$
  where *T^t^* is the chaotic number produced by the Tent mapping.

(7) If the exit condition is satisfied, the algorithm terminates; otherwise, it proceeds to (2) to continue the cycling.

**Figure 1:**
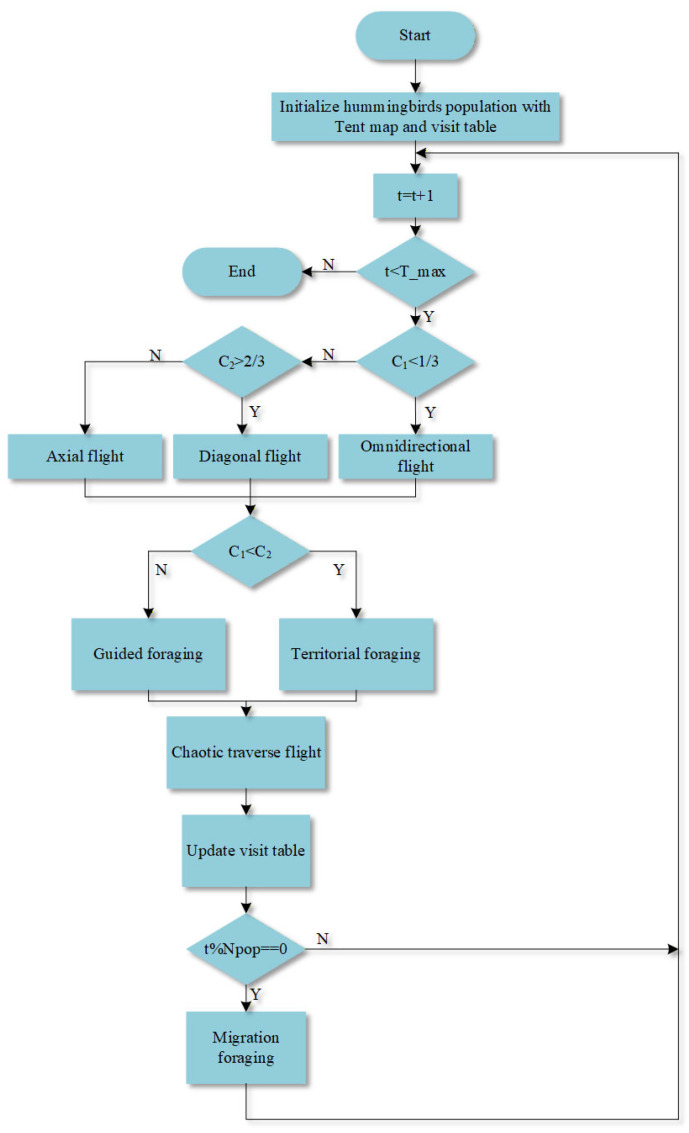
The workflow diagram of CAHA.

The overall algorithmic process of CAHA is presented in Figure 1.

## 3. Simulations

### 3.1. Process of Simulation

To validate the feasibility and validity of the CAHA algorithm, temperature inversion simulations were carried out using six materials (A–F) featuring different emissivity trends. The emissivities of the six materials originated from Reference [14] and their distributions are presented in Table 1.

The emissivity distributions of these six materials are highly representative and can encompass the distribution trends of a significant portion of materials. The number of multispectral channels is 8, with the effective wavelengths being 0.4 µm, 0.5 µm, 0.6 µm, 0.7 µm, 0.8 µm, 0.9 µm, 1.0 µm, and 1.1 µm respectively. The blackbody reference temperature is 1600 K and the true temperature is 1800 K. During the simulation, the CAHA algorithm was compared with the algorithms in References [16,17] under 0 noise and 5% random noise conditions. The results indicate that CAHA holds more advantages in both time and accuracy and possesses a strong anti-noise performance. Simultaneously, the relationship between the number of repeated runs of CAHA and the optimal solution was examined, and a conclusion regarding the application feasibility of CAHA in practical engineering problems was reached.

### 3.2. Results Analysis

**Figure 2:**
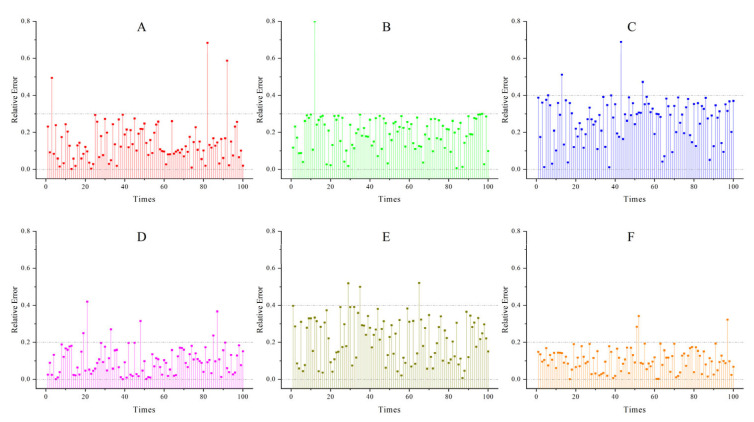
Relative Errors of the CAHA algorithm used by six noise-free models.

**Figure 3:**
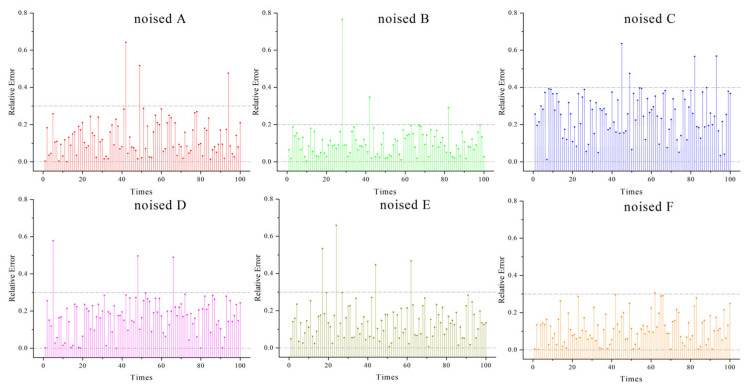
Relative Errors of the CAHA algorithm used by six noisy models.

**Accuracy**: The simulation experiments compared the relative errors of the inversion results of CAHA and the algorithms in [16,17] under noise-free and 5% noise conditions. As can be seen from Table 2, CAHA exhibited superior performance in the simulation experiments. In the 100 inversions of the six emissivity models under noise-free voltage signals, the average relative error of CAHA was no greater than 0.24% (Figure 2 shows the errors of 100 noise-free inversions. Table 3 presents the optimal solutions obtained from 100 inversions for six models), and in the 100 inversions with 5% noise added, the average relative error did not exceed 0.25% (Figure 3 shows the errors of 100 inversions with 5% noise. Table 4 presents the optimal solutions obtained from 100 inversions for six models with 5% noise).

To ensure a fair comparison, all three algorithms were evaluated on the same computer under identical CPU load conditions. (Simulation environment: PyCharm 2023.1, Python 3.9; Intel Core i7-13700K CPU at 3.40 GHz; 64 G RAM).

The single inversion times for the three algorithms are listed in Table 5. As can be observed from Table 5, the CAHA algorithm requires only approximately 0.15 s per run, outperforming the other two algorithms in terms of time performance, thereby sufficiently meeting the requirements of practical engineering applications.

**Figure 4:**
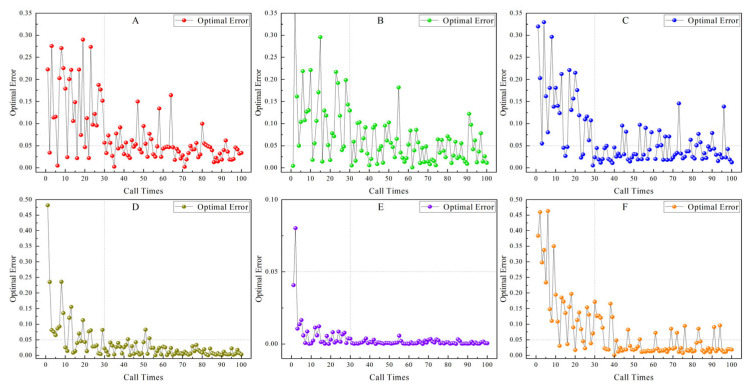
The relationship between the Call Times (ranging from 1 to 100 times) and the Optimal Error.

Meanwhile, superior time performance can remedy the issue of falling into local optima due to the random initial solution group of meta-heuristic algorithms through multiple operations. That is, the optimal error is selected as the output of the optimal solution through multiple operations. Figure 4 is the curve graph of the numerical value of the optimal relative error and the optimal fitness from 10 to 100 times of the simulation inversion operation of six emission rate models with 5% noise using the CAHA algorithm.

**Figure 5:**
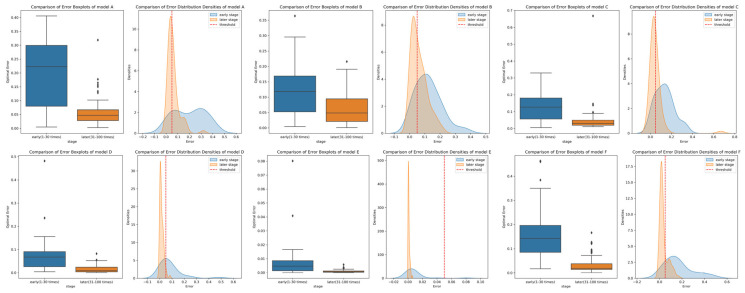
Comparison of error boxplots and distribution densities of six models.

As Figure 5 and Table 6 explored in the simulation performance analysis of the CAHA algorithm with six models, during its execution of 1–100 inversion operations, Mann–Whitney U test-based statistical analysis uncovers an overall negative correlation between the optimal error and operation times. Remarkably, post 30 operations, the optimal errors of the six models (A–C) all decline beneath 0.05%.

This reveals that 30 operations strike a balance between computational time and precision. Building on this insight from the simulation-based analysis, when transitioning to experimental calculations, employing 30 CAHA algorithm operations to screen for the minimum error as output becomes a logical bridge (due to the excellent time efficiency of the CAHA algorithm, a single-inversion operation takes only 0.15 s, and 30 consecutive inversions require merely 4.5 s in total), ensuring the algorithm’s application in experiments benefits from this carefully identified operational parameter.

### 3.3. Offline Testing

To validate the feasibility of the CAHA algorithm in practical applications, the temperature inversion of the rocket nozzle in [14] was utilized for the verification experiment. In [14], the temperature of the rocket nozzle (with a designed temperature of 2490 K) was measured using an 8-channel multispectral thermometer, and the voltages of each channel of the Multiple Wavelength Pyrometer (MWP) and the corresponding wavelengths were calibrated with 2252 K as the reference temperature (see Table 7).

Measurements were conducted at 12 consecutive time points on the rocket nozzle, and the voltage values of each channel obtained are presented in Table 8.

The algorithm parameter settings were identical to those in the simulation experiment, with the upper and lower bounds of the solution population set at 0.3 and 0.7, respectively. Based on the simulation results of the previous section, the approach of conducting 30 operations and taking the optimal fitness was adopted for the inversion. The inversion results are presented in Table 9.

The inversion results indicate that the CAHA algorithm possesses a relatively high inversion precision. Through the method of obtaining the optimal result from 30 inversions, the average relative error of the 30 inversions does not exceed 0.48% and the optimal relative error does not exceed 0.054%. The absolute error does not exceed 0.74 K, the single operation time is less than 0.156 s and the total operation time of 30 times does not exceed 4.69 s. Hence, as a novel and more efficient inversion algorithm, CAHA offers a fresh perspective for the inversion of multi-band temperature measurement data, namely, extracting the optimal result from multiple operations. This approach can satisfy the real-time requirements of the vast majority of engineering applications, while achieving exceptionally outstanding inversion accuracy.

## 4. Experiment

### 4.1. Temperature Calibration Experiment

For the actual measurement experiment, a 25-channel multispectral camera was used for temperature measurement verification (8 bands were selected for retrieval in the experiment), and the parameters of the multispectral camera are listed in Table 10. Prior to the experiment, a standard blackbody source was utilized to calibrate the quantitative relationship between the radiance (R) of the multispectral camera and the image pixel gray level (G).

**Figure 6:**
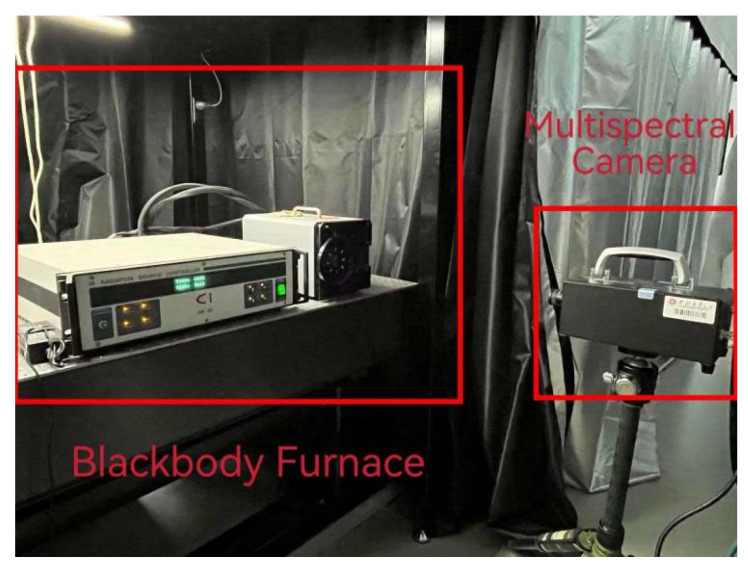
Calibration experiment setup diagram.

**Figure 7:**
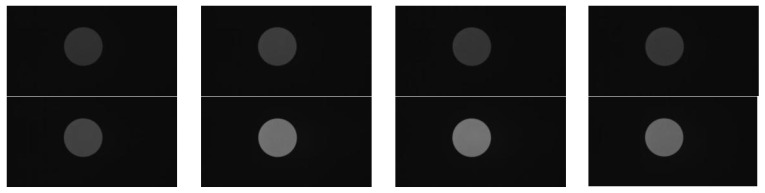
8-channel blackbody source gray-scale images.

In this study, an SR20-32 medium-temperature blackbody furnace was adopted as the standard radiant blackbody source. This blackbody furnace has a temperature range of 50–1000 °C with an accuracy of ±0.1 °C and a spectral emissivity range of 0.99 ± 0.01. During the experiment, the gray values displayed by the camera sensor were recorded by adjusting the blackbody temperature. The experimental setup is shown in Figure 6, and the 8-channel blackbody radiation gray-scale images obtained from the experiment are presented in Figure 7.

The set temperature range of the blackbody is 800–1000 °C with a step size of 50 °C. The multispectral camera selects the channel images at 658.88 nm, 700.34 nm, 739.31 nm, 780.66 nm, 814.62 nm, 852.64 nm, 889.84 nm, and 921.46 nm respectively for temperature and emissivity retrieval experiments.

The average gray level of the 20 × 20 region of effective pixel gray levels at the center of the image is selected as the pixel gray level (G) corresponding to the standard blackbody radiation. The average gray levels under different temperatures and bands are listed in Table 11.

### 4.2. Experiment on Temperature Inversion of Blackbody Sources

**Figure 8:**
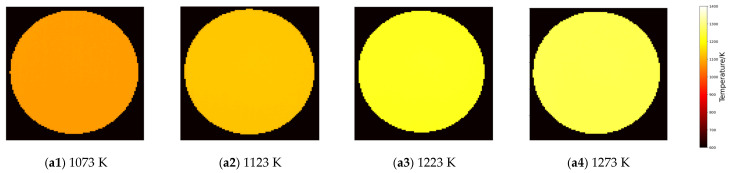

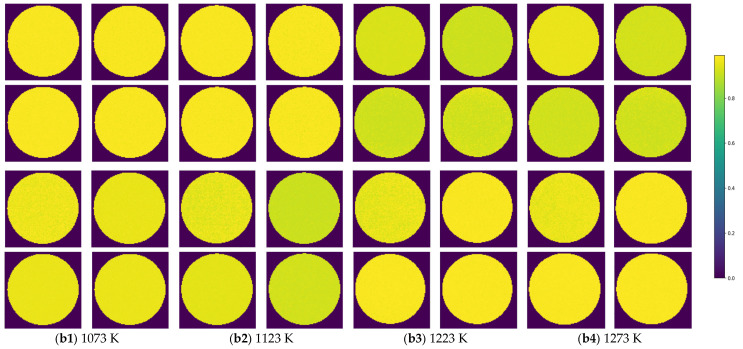
Results of retrieved temperature (**a1**–**a4**) and emissivity (**b1**–**b4**) of the standard blackbody at 1073 K, 1123 K, 1223 K, and 1273 K under the reference temperature of 1173 K.

To preliminarily verify the feasibility of the algorithm in practical applications, this section adopts a standard blackbody source for temperature retrieval. A temperature of 900 °C (1173 K) is used as the reference temperature and multispectral gray-scale images at 800 °C (1073 K), 850 °C (1123 K), 950 °C (1223 K), and 1000 °C (1273 K) are retrieved respectively. Since it is a standard blackbody source, the algorithm accuracy can be verified by comparing the standard temperature/emissivity with the retrieved temperature/emissivity. Figure 8 presents the retrieved results at four different temperature points, where subfigure (a) shows the reconstructed two-dimensional temperature distribution, and subfigure (b) shows the emissivity distribution images corresponding to the eight channels.

From the retrieval of temperature and emissivity based on calibration data at different temperatures, the absolute errors (AE) and relative errors (RE) between the average temperature/average emissivity of the retrieval results and the actual values are presented in Table 12 and Table 13.

It can be seen from Table 12 that the absolute error (AE) of temperature retrieval of the CAHA algorithm for the standard blackbody source is no more than 12 K and the relative error (RE) is no more than 0.93%. It can be seen from Table 13 that the absolute error of emissivity retrieval is no more than 0.0076 and the relative error is no more than 0.77%, which is consistent with the conclusions obtained from the aforementioned simulation experiments. In contrast, the IGWO algorithm, as shown in Table 14 and Table 15, yields a maximum temperature relative error of 1.36% and a maximum emissivity relative error of 4.77%, both significantly higher than those of CAHA. This performance discrepancy can be attributed to the fact that IGWO performs only a single inversion and, despite its shorter execution time, is prone to falling into local optima due to the random initialization of its population. In contrast, CAHA enhances population diversity through chaotic mapping and selects the optimal result from multiple independent runs, thereby improving both accuracy and robustness.

### 4.3. Uncertainty Analysis

Based on Equation (4), all components affecting the uncertainty in retrieved temperature and emissivity can be summarized as follows:

Δλ_i_: uncertainty in the effective wavelength λi;

G_Vi_: uncertainty in the measured signal Vi of the sample in the i-th channel;

G_Vib_: uncertainty in the reference signal Vib of the blackbody in the i-th channel;

ΔT: random uncertainty in the true temperature T of the sample (set by the blackbody furnace);

ΔT_b_: random uncertainty in the reference temperature Tb of the blackbody.

Incorporating all parameters influencing the uncertainty into the random uncertainty, the following model can be established according to Equation (4):

(15) $$\frac{V_{\lambda_{i}}\pm G_{V_{\lambda_{i}}}}{V_{\lambda_{ib}}\pm G_{V_{\lambda_{i_{b}}}}}=\varepsilon \left(\lambda_{i}\pm \Delta \lambda_{i},T\pm \Delta T\right)e^{\frac{c_{2}}{\lambda_{i}\pm \Delta \lambda_{i}}\left(\frac{1}{T_{b}\pm \Delta T_{b}}-\frac{1}{T\pm \Delta T}\right)}.$$

To evaluate the uncertainty in the algorithm-retrieved temperature and emissivity, a Monte Carlo method was employed to quantify the contributions of individual components to the overall uncertainty, with M = 10^4^ trials. The experimental results are presented in Table 16 and Table 17.

As shown in Table 14, the uncertainty contributions from the camera’s grayscale-to-radiance response to the blackbody furnace G_Vi_ and the reference signal of the blackbody furnace G_Vib_ are the largest among all considered factors, reaching up to 0.451 K and 0.529 K, respectively, under typical conditions. Furthermore, the disturbances in the lower temperature bands are greater than those in the higher temperature bands, which is attributed to the better response of the experimental camera in the higher temperature range.

From Table 15, it can be observed that the perturbations of the aforementioned parameters lead to a maximum single contribution to emissivity uncertainty of 0.1416%. Overall, the combined uncertainties for the retrieved temperature and emissivity are 2.6 K and 0.63%, respectively.

### 4.4. Experiment on Temperature Inversion of Candle Flame

**Figure 9:**
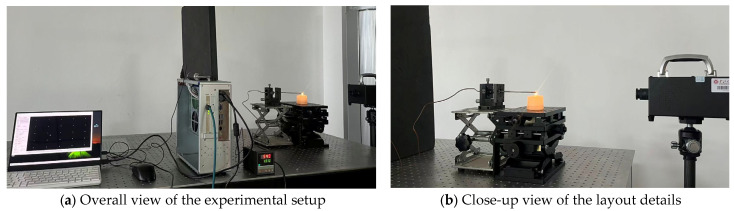
Experimental layout of the candle flame.

**Figure 10:**
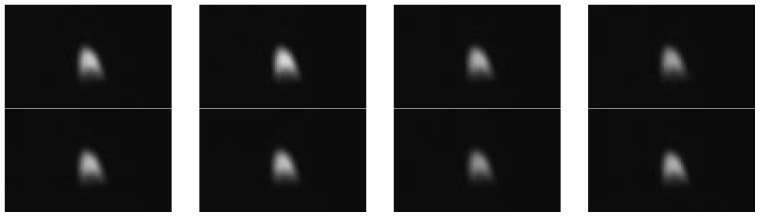
8-channel grayscale images of candle flame.

**Figure 11:**
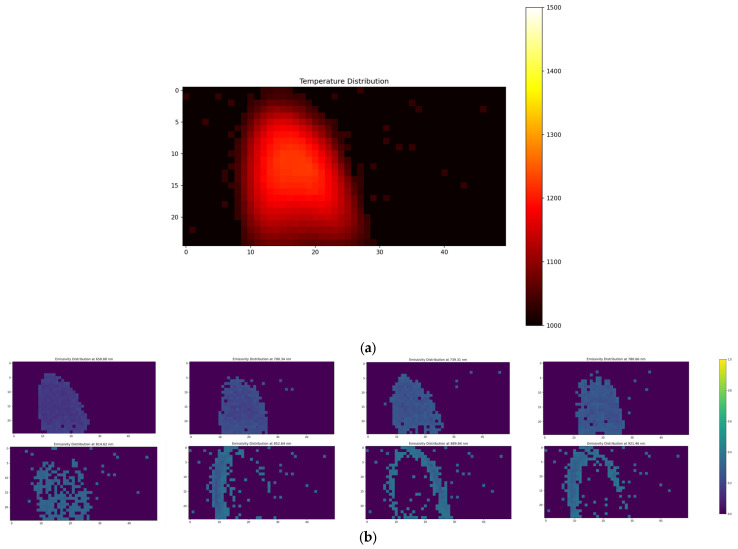
Results of candle flame retrieved temperature (**a**) and emissivity (**b**).

Based on the aforementioned calibration data, this section conducts a practical experiment on the two-dimensional temperature/emissivity retrieval of candle flames using the CAHA algorithm. A reference temperature of 700 °C (973 K) is adopted, and a thermocouple is used to measure and verify the highest temperature point of the candle’s outer flame. The experimental setup is shown in Figure 9, the grayscale images of the paraffin candle flame captured by the multispectral camera are shown in Figure 10, and the retrieved temperature/emissivity distribution images are presented in Figure 11.

From the aforementioned retrieval results, it can be concluded that the retrieved maximum temperature of the flame’s outer cone is 976 K, while the maximum temperature of the outer cone measured by the thermocouple is 969.58 K. The absolute error is 6.42 K, and the relative error is 0.66%. The average retrieved emissivity across different wavelength bands is presented in Table 18. For comparison, the IGWO algorithm yields a maximum temperature of 982.34 K under the same conditions, corresponding to an absolute error of 12.76 K and a relative error of 1.32%, with the retrieved emissivity results listed in Table 19. The higher accuracy achieved by CAHA further demonstrates the advantage of its multi-run optimization strategy combined with chaotic population initialization, which effectively mitigates the risk of converging to suboptimal solutions—a limitation commonly encountered in single-run metaheuristic approaches such as IGWO.

## 5. Conclusions

This paper proposes a multi-band temperature measurement data retrieval method based on the chaotic artificial hummingbird algorithm (CAHA), which has the following characteristics:

(1) Based on a reference temperature model, it does not require predefining the emissivity;
(2) It automatically generates an initial solution population through chaotic mapping, avoiding manual intervention;
(3) It exhibits high retrieval accuracy and stability in simulations and offline tests of rocket nozzles;
(4) The effectiveness and reliability of the method are further verified in experimental validation, and it has the potential for practical application.

## Figures and Tables

**Table 1:** Emissivity Model.

	0.4 µm	0.5 µm	0.6 µm	0.7 µm	0.8 µm	0.9 µm	1.0 µm	1.1 µm
A	0.85	0.75	0.67	0.60	0.55	0.50	0.48	0.45
B	0.45	0.48	0.50	0.55	0.60	0.67	0.75	0.85
C	0.45	0.55	0.65	0.75	0.74	0.65	0.55	0.45
D	0.85	0.75	0.65	0.55	0.54	0.65	0.75	0.80
E	0.85	0.65	0.55	0.65	0.84	0.65	0.55	0.50
F	0.50	0.55	0.65	0.84	0.64	0.55	0.65	0.85

**Table 2:** The average relative errors of the three algorithms in the simulation experiments (with 100 inversions).

	BFGS		IGWO		CAHA	
Noise	0%	5%	0%	5%	0%	5%
A	0.41	\	0.17	0.14	0.14	0.13
B	0.09	\	0.05	0.03	0.19	0.10
C	0.29	\	0.12	0.25	0.24	0.25
D	0.14	\	0.08	0.23	0.10	0.17
E	0.01	\	0.18	0.33	0.22	0.14
F	0.35	\	0.11	0.22	0.10	0.11

**Table 3:** The optimal solution results of 100 inversions with 0% noise.0

		0.4 µm	0.5 µm	0.6 µm	0.7 µm	0.8 µm	0.9 µm	1.0 µm	1.1 µm
A	Emissivity	0.8528	0.7508	0.6708	0.5989	0.5499	0.5002	0.4803	0.4496
	Temperature/K	1799.70	1799.88	1799.84	1800.29	1800.04	1799.92	1799.88	1800.22
B	Emissivity	0.4590	0.4872	0.5043	0.5548	0.6050	0.6755	0.7552	0.8553
	Temperature/K	1798.22	1798.33	1798.86	1798.62	1798.50	1798.35	1798.46	1798.45
C	Emissivity	0.4520	0.5511	0.6510	0.7503	0.7406	0.6504	0.5506	0.4503
	Temperature/K	1800.27	1799.94	1799.76	1799.96	1800.02	1799.92	1799.99	1799.78
D	Emissivity	0.8455	0.7514	0.6498	0.5506	0.5398	0.6506	0.7499	0.8004
	Temperature/K	1800.48	1799.78	1800.03	1799.84	1800.07	1799.83	1800.00	1799.89
E	Emissivity	0.8498	0.6513	0.5509	0.6505	0.8403	0.6502	0.5505	0.5002
	Temperature/K	1800.02	1799.78	1799.77	1799.88	1799.93	1799.94	1799.80	1799.88
F	Emissivity	0.4997	0.5513	0.6511	0.8389	0.6503	0.5503	0.6496	0.8490
	Temperature/K	1799.37	1800.41	1800.15	1800.48	1800.03	1800.20	1800.05	1799.95

**Table 4:** The optimal solution results of 30 inversions with 5% noise.

		0.4 µm	0.5 µm	0.6 µm	0.7 µm	0.8 µm	0.9 µm	1.0 µm	1.1 µm
A	Emissivity	0.8412	0.7391	0.6041	0.5649	0.5539	0.4629	0.4677	0.4720
	Temperature/K	1800.32	1799.73	1800.34	1800.23	1799.58	1799.75	1799.78	1800.32
B	Emissivity	0.4325	0.4697	0.5353	0.5580	0.6623	0.7233	0.7713	0.8885
	Temperature/K	1797.59	1800.49	1800.71	1799.26	1798.36	1801.48	1799.72	1801.84
C	Emissivity	0.4572	0.5545	0.6887	0.7675	0.7729	0.5853	0.5752	0.4431
	Temperature/K	1799.71	1799.91	1799.58	1799.79	1799.82	1799.89	1799.67	1799.82
D	Emissivity	0.8783	0.7452	0.6642	0.5058	0.5271	0.6312	0.7221	0.7531
	Temperature/K	1801.41	1798.70	1799.75	1799.65	1798.93	1801.09	1800.52	1800.00
E	Emissivity	0.7944	0.7945	0.5477	0.6336	0.7212	0.6533	0.4832	0.5057
	Temperature/K	1800.33	1800.04	1800.03	1800.14	1800.31	1800.05	1799.96	1800.10
F	Emissivity	0.5035	0.5480	0.6493	0.8374	0.6499	0.5495	0.6499	0.8502
	Temperature/K	1799.37	1800.41	1800.15	1800.48	1800.43	1800.20	1800.05	1799.95

**Table 5:** Comparison of algorithm running times.

	BFGS	IGWO	CAHA
Running Time/s	0.2	0.28	0.15

**Table 6:** Error median distribution in different stages and Mann–Whitney U Test outcomes.

		A	B	C	D	E	F
Error Median (%)	(1st–30th time)	0.2224	0.1181	0.1273	0.0675	0.0047	0.1429
	(31st–100th time)	0.0472	0.0484	0.0305	0.0086	0.0007	0.0189
Mann–Whitney U Test		417.0	552.0	352.0	303.0	427.0	191.0
*P*		0.0000	0.0001	0.0000	0.0000	0.0000	0.0000
Conclusions		The null hypothesis is rejected at the significance level of 0.05.					
		In the later stage (from the 31st to the 100th time), the error distribution is significantly lower than that in the early stage (1st–30th time).					

**Table 7:** Effective wavelength and output of the pyrometer at the reference temperature.

Channels	1	2	3	4	5	6	7	8
*λ_i_*/µm	0.574	0.592	0.623	0.654	0.698	0.748	0.826	0.914
*V_i_*/mV	39.4	139.7	117.5	363.7	345.0	493.9	320.7	406.7

**Table 8:** Practical data performed on a solid propellant rocket plume.

	*V_i_* of 8 Channels							
Measuring Times	1	2	3	4	5	6	7	8
1	46.3	254.1	165.3	481.5	367.8	495.0	273.7	323.5
2	46.3	254.1	170.2	476.6	372.7	500.0	278.6	328.4
3	46.3	244.2	165.3	471.6	362.8	495.0	268.7	323.5
4	46.3	244.2	170.2	481.5	372.7	509.9	283.6	333.4
5	46.3	249.1	160.3	471.6	362.8	490.1	268.7	318.5
6	46.3	244.2	160.3	461.7	352.9	480.2	253.9	303.7
7	41.3	234.3	155.4	456.8	343.0	465.3	248.9	293.8
8	46.3	244.2	160.3	461.7	352.9	475.2	253.9	298.7
9	41.3	239.2	155.4	461.7	343.0	470.3	253.9	303.7
10	41.3	239.2	155.4	456.8	343.0	470.3	248.9	298.7
11	41.3	234.3	155.4	451.8	333.1	460.4	239.0	293.8
12	41.3	239.2	155.4	456.8	343.0	470.3	248.9	298.7

**Table 9:** Temperature inversion result by CAHA algorithm.

Times Point	1	2	3	4	5	6
Inversion/K	2489.67	2490.29	2488.66	2490.13	2489.26	2489.88
Absolute Error/K	0.33	0.29	1.34	0.13	0.74	0.12
Minimum Error/%	0.0134	0.0117	0.0540	0.0050	0.0300	0.0046
Average Relative Error (30 times)/%	0.326	0.277	0.472	0.274	0.327	0.195
Total Time/s	4.56	4.56	4.55	4.69	4.68	4.67
Time per Run/s	0.152	0.152	0.152	0.156	0.156	0.156
**Times Point**	**7**	**8**	**9**	**10**	**11**	**12**
Inversion/K	2489.86	2490.06	2489.99	2490.02	2489.28	2490.12
Absolute Error/K	0.14	0.06	0.01	0.02	0.72	0.12
Minimum Error/%	0.0055	0.0022	0.00003	0.0010	0.0288	0.0048
Average Relative Error (30 times)/%	0.310	0.122	0.117	0.186	0.456	0.169
Total Time/s	4.58	4.56	4.58	4.57	4.64	4.67
Time per Run/s	0.153	0.152	0.153	0.152	0.155	0.156

**Table 10:** Multispectral camera parameters and performance indicators.

Parameters and Performance Indicators	Multispectral Radiometric Sensor
Instrument Model	HYM20X8-HS25 (Tianjin Huapu Shiwei Technology Co., Ltd., Tianjin, China)
Sensor Type	Research-Grade CMOS
Sensor Resolution	2048 × 1088
Sensor Frame Rate	340 fps@All channels Full-pixel 10-Gi Port
Spectral Chanel Count	25 Channels
Spectral Bandwidth	10 nm per Channel
Response Range	≥665~975 nm
Sampling Bit Depth	12 bit
Radiometric Measurement Range	800~2700 °C

**Table 11:** Average grayscale values at different temperatures and wavelengths.

	*G_i_* of 8 Channels							
Blackbody Temperature /K	658.88 nm	700.34 nm	739.31 nm	780.66 nm	814.62 nm	852.64 nm	889.84 nm	921.46 nm
1073	232.03	245.60	230.50	235.60	251.15	326.57	338.90	320.85
1123	247.20	265.73	247.03	253.05	274.35	374.00	385.88	363.50
1173	357.90	415.55	364.65	378.95	446.47	716.50	752.39	678.78
1223	403.41	476.53	422.32	436.98	516.64	826.50	853.25	769.54
1273	756.70	949.82	816.18	843.25	1048.2	1759.7	1835.0	1603.6

**Table 12:** Absolute errors (AE) and relative errors (RE) of the temperature retrieved by the CAHA algorithm for blackbody sources at different temperatures.

	Average Inversion Temperature/K	AE /K	RE /%
1073 K	1080.39	7.39	0.69
1123 K	1128.72	5.72	0.51
1223 K	1222.31	0.69	0.05
1273 K	1261.14	11.86	0.93

**Table 13:** Retrieved average emissivity results using the CAHA algorithm at different temperatures and in different bands.

	1073 K	1123 K	1223 K	1273 K
658.88 nm	0.9903	0.9900	0.9770	0.9870
700.34 nm	0.9890	0.9868	0.9712	0.9894
739.31 nm	0.9882	0.9899	0.9758	0.9826
780.66 nm	0.9798	0.9874	0.9855	0.9800
814.62 nm	0.9864	0.9722	0.9861	0.9899
852.64 nm	0.9783	0.9796	0.9901	0.9906
889.84 nm	0.9763	0.9766	0.9912	0.9925
921.46 nm	0.9770	0.9781	0.9922	0.9930
Average	0.9832	0.9826	0.9824	0.9881
AE	0.0068	0.0074	0.0076	0.0019
RE/%	0.69	0.75	0.77	0.19

**Table 14:** Absolute errors (AE) and relative errors (RE) of the temperature retrieved by the IGWO algorithm for blackbody sources at different temperatures.

	Average Inversion Temperature/K	AE /K	RE /%
1073 K	1063.90	9.10	0.85
1123 K	1130.52	7.52	0.67
1223 K	1213.46	9.54	0.78
1273 K	1290.37	17.37	1.36

**Table 15:** Retrieved average emissivity results using the IGWO algorithm at different temperatures and in different bands.

	1073 K	1123 K	1223 K	1273 K
658.88 nm	0.9523	0.9728	0.9781	0.9387
700.34 nm	0.9516	0.9719	0.9829	0.9412
739.31 nm	0.9508	0.9707	0.9763	0.9423
780.66 nm	0.9497	0.9698	0.9817	0.9431
814.62 nm	0.9485	0.9689	0.9795	0.9448
852.64 nm	0.9472	0.9676	0.9832	0.9452
889.84 nm	0.9461	0.9665	0.9778	0.9469
921.46 nm	0.9454	0.9653	0.9804	0.9473
Average	0.9490	0.9692	0.9800	0.9437
AE	0.041	0.0208	0.0100	0.0462
RE/%	4.10	2.10	1.01	4.77

**Table 16:** Contribution of each parameter to the temperature uncertainty.

	1073		1123		1223		1273	
σ*T* (K)	658.88 nm	921.46 nm	658.88 nm	921.46 nm	658.88 nm	921.46 nm	658.88 nm	921.46 nm
Δλ_i_	0.189	0.301	0.164	0.278	0.045	0.082	0.044	0.092
ΔT_b_	0.109	0.225	0.089	0.167	0.048	0.118	0.077	0.123
G_Vi_	0.343	0.451	0.238	0.311	0.219	0.299	0.278	0.325
G_Vib_	0.378	0.529	0.324	0.425	0.252	0.293	0.257	0.306

**Table 17:** Contribution of each parameter to the emissivity uncertainty.

	1073		1123		1223		1273	
Σ*ε* (%)	658.88 nm	921.46 nm	658.88 nm	921.46 nm	658.88 nm	921.46 nm	658.88 nm	921.46 nm
Δλ_i_	0.0146	0.0228	0.0258	0.0427	0.0026	0.0113	0.0189	0.0272
ΔT_b_	0.0170	0.0317	0.0198	0.0276	0.0073	0.0115	0.0182	0.0494
G_Vi_	0.0736	0.1381	0.0563	0.1163	0.0419	0.0873	0.0380	0.0693
G_Vib_	0.0687	0.1416	0.0872	0.1351	0.0574	0.0701	0.0763	0.0858

**Table 18:** Retrieved emissivity results of candle flames across different wavelength bands using the CAHA Algorithm.

Wavelength	Emissivity	Wavelength	Emissivity
658.88 nm	0.2106	814.62 nm	0.3416
700.34 nm	0.2606	852.64 nm	0.3831
739.31 nm	0.2965	889.84 nm	0.3912
780.66 nm	0.3020	921.46 nm	0.3867

**Table 19:** Retrieved emissivity results of candle flames across different wavelength bands using the IGWO algorithm.

Wavelength	Emissivity	Wavelength	Emissivity
658.88 nm	0.3917	814.62 nm	0.4271
700.34 nm	0.3752	852.64 nm	0.3945
739.31 nm	0.4183	889.84 nm	0.4028
780.66 nm	0.3869	921.46 nm	0.3816

## Data Availability

The original contributions presented in this study are included in the article. Further inquiries can be directed to the corresponding authors.

## Abbreviations

The following abbreviations are used in this manuscript:

CAHA	Chaotic Artificial Hummingbird Algorithm
AE	Absolute Error
RE	Relative Error
